# A comparative analysis of differentially expressed mRNAs, miRNAs and circRNAs provides insights into the key genes involved in the high-altitude adaptation of yaks

**DOI:** 10.1186/s12864-021-08044-9

**Published:** 2021-10-15

**Authors:** Qianyun Ge, Yongbo Guo, Wangshan Zheng, Yuan Cai, Xuebin Qi, Shengguo Zhao

**Affiliations:** 1 College of Animal Science and Technology, Gansu Agricultural University, Lanzhou, 730070 China; 2 State Key Laboratory of Genetic Resources and Evolution, Kunming Institute of Zoology, Chinese Academy of Sciences, Kunming, 650223 China

**Keywords:** Yak, Zaosheng cattle, Transcriptome, Hypoxia, Lung

## Abstract

**Background:**

Yaks that inhabit the Tibetan Plateau exhibit striking phenotypic and physiological differences from cattle and have adapted well to the extreme conditions on the plateau. However, the mechanisms used by these animals for the regulation of gene expression at high altitude are not fully understood.

**Results:**

Here, we sequenced nine lung transcriptomes of yaks at altitudes of 3400, 4200 and 5000 m, and low-altitude Zaosheng cattle, which is a closely related species, served as controls. The analysis identified 21,764 mRNAs, 1377 circRNAs and 1209 miRNAs. By comparing yaks and cattle, 4975 mRNAs, 252 circRNAs and 75 miRNAs were identified differentially expressed. By comparing yaks at different altitudes, we identified 756 mRNAs, 64 circRNAs and 83 miRNAs that were differentially expressed (fold change ≥2 and *P*-value < 0.05). The pathways enriched in the mRNAs, circRNAs and miRNAs identified from the comparison of yaks and cattle were mainly associated with metabolism, including ‘glycosaminoglycan degradation’, ‘pentose and glucuronate interconversions’ and ‘flavone and flavonol biosynthesis’, and the mRNAs, circRNAs and miRNAs identified from the comparison of yaks at different altitude gradients were significantly enriched in metabolic pathways and immune and genetic information processing pathways. The core RNAs were identified from the mRNA-miRNA-circRNA networks constructed using the predominant differentially expressed RNAs. The core genes specific to the difference between yaks and cattle were associated with the endoplasmic reticulum and fat deposition, but those identified from the comparison among yaks at different altitude gradients were associated with maintenance of the normal biological functions of cells.

**Conclusions:**

This study enhances our understanding of the molecular mechanisms involved in hypoxic adaptation in yaks and might contribute to improvements in the understanding and prevention of hypoxia-related diseases.

**Supplementary Information:**

The online version contains supplementary material available at 10.1186/s12864-021-08044-9.

## Background

The mechanisms regulating the adaptation of organisms to high-altitude environments have become topics of great interest in recent years. Yaks (*B. grunniens*) are an iconic symbol of the Tibetan Plateau. Currently, there are more than 15 million yaks living on the Tibetan Plateau, accounting for about 90% of the world’s yak population. Yaks are indispensable to Tibetans and furnish primary resources [1], which makes yaks an ideal model for studying adaptation to plateau environments. Yaks have evolved some anatomical and physiological traits that allow them to survive in extreme high-altitude environments, and these traits include larger lungs and hearts [2], blunted hypoxic pulmonary vasoconstriction [3], a strong environmental sense [2] and high energy metabolism [4]. Extensive and in-depth investigations of high-altitude adaptation have been performed at the levels of morphology [5], anatomy [6], hemodynamics [6], physiology [7] and genomics [1, 8]. Because the cattle reared in the yak habitat suffers from pulmonary hypertension [9–11], the comparison of yaks and cattle contributes to an improved understanding of evolutionary adaptation to high altitudes [3, 4, 12]. Tibetans have also acquired similar physiological features, which helps to provide more efficient blood flow and oxygen under hypobaric hypoxia [13]. In addition, compared with the lowland cattle and lowland Han populations, the hemoglobin levels of yaks and Tibetans are maintained at normal levels [14, 15], which suggests that yaks and Tibetans likely utilize similar strategies to protect themselves from high-altitude polycythemia. Previous studies have identified *EPAS1* and *EGLN1* as two key genes for maintaining normal levels of hemoglobin concentrations under hypoxic environment [15, 16], whereas the genes for yaks to adapt to hypoxia seem to be different from humans [17]. Compared with yaks, humans are more recent inhabitants on the Tibetan Plateau [18–21]. Therefore, natural selection was likely to affect different set of genes and pathways in yaks.

Accumulating evidence strongly implicates noncoding RNAs (ncRNAs), particularly microRNAs (miRNAs) [22], long noncoding RNAs (lncRNAs) [23–25], and circular RNAs (circRNAs) [26], in gene expression. In a previous study, we investigated the lung expression patterns of dysregulated lncRNAs in yak and cattle models and demonstrated that lncRNAs contribute to yak hypoxia. To further investigate the regulatory role of ncRNAs in hypoxia, we focused on circRNAs, a class of ncRNAs that can regulate transcriptional and posttranscriptional gene expression [27]. Unlike linear RNAs, circRNAs consist of covalently closed continuous loops without 5′-3′ polarity and a poly (A) tail and might function as microRNA sponges to modulate the expression of parental genes through competing endogenous RNA (ceRNA) networks [28]. Therefore, we hypothesized that circRNAs, as a new regulatory layer, might play an important role in yak hypoxia.

Lung plays an important role in the adaptation to hypoxia in the plateau environment as the respiratory system. To identify the mechanisms that regulate the adaptation to hypoxia, we used a microarray analysis approach to identify the profiles of differentially expressed circRNAs, miRNAs and mRNAs in the lung of yaks. Since the yak is a native species on the plateau, these animals are only located at altitudes between 3000 and 5000 m above sea level, and thus, we cannot use a lowland control. Yaks and cattle constitute a pair of closely related species that diverged 5 million years ago [1]. Although yaks and cattle are different species, their genomes exhibit strong similarities, including an identical number of chromosomes (30 chromosomes), similar karyotypes [2] and extensive synteny [1], and thus, these two species can be used to decipher the mechanisms underlying adaptation to hypoxia. Genetic of mammalian adaptation can be based on genomic comparisons between closely related species [29, 30]. To further explore the changes in the regulation of genes related to hypoxic adaptation that occur in yak lung tissue at increasing altitudes, lung samples from yaks at different altitudes were also subjected to a microarray analysis. We then performed Gene Ontology (GO) and Kyoto Encyclopedia of Genes and Genomes (KEGG) analyses and constructed a circRNA-associated ceRNA network. The findings from this study will expand our understanding of the potential role of the circRNA-associated ceRNA network in hypoxia in the yak lung.

## Methods

### Experimental animals and sample collection

Lung tissue were collected from indigenous adult male yaks at three different altitudes (3400 m, 4200 m and 5000 m) in three biological replicates per altitude, two indigenous adult male Zaosheng cattle at an altitude of 1500 m as a lowland control. The location and other details of samples were provided in the Table S1 and Fig. S1. We chose animals of breeding age because of their relatively stable gene expression. Prior to sample collection, ensure the health of the animals. The yaks were humanely sacrificed with the following procedures: Electrically stunned (120 V dc, 12 s) prior to exsanguination, sacrificed while in the coma by bloodletting from carotid artery and jugular vein, and dissected rapidly to obtain lung tissue samples. 10G of data were generated from each sample.

### Read mapping and transcript assembly

Raw data were filtered using the following series of steps to remove low-quality data for subsequent analysis. Low-quality reads were removed by using BWA algorithm [31]. Subsequently, low-quality reads, reads without 3′ adapters, unknown base calls (N) were trimmed from the raw miRNA data. Mapping to the reference genome (ftp://ftp.ncbi.nlm.nih.gov/genomes/refseq/vertebrate_mammalian/Bos_mutus/annotation_releases/current/GCF_000298355.1_BosGru_v2.0/GCF_000298355.1_BosGru_v2.0_genomic.fna.gz) based on the HISAT2 software (v2.0.5). The generated bam files were sent to StringTie (v1.3.2d), and the information was then merged to obtain the assembly results.

### Identification of circRNAs and miRNAs

The back-splice algorithm and FindCirc software were used to identify the junctions of unmapped reads and the prediction of the circRNAs, respectively [32].

To filter out other small RNA species, the unique reads were aligned to the Rfam RNA family database in the miRNA analysis. The BLAST parameters were as follows: (1) E value not higher than 1; (2) score ≥ 15 (at least 15 bases); and (3) no more than one mismatched base. The miRNA of this species were compared using the miRBase database [33]. Identify known miRNAs from the sequencing data by comparing with the miRBase database. Determine the location of novel miRNAs in the reference genome using Bowtie2 [34], and MIREAP was then used for the prediction of novel miRNAs based on the localization results.

### Quantification and differential analysis of RNA expression levels

The previous analysis revealed the read counts of each sample. FPKM (fragments per kilobase of transcript per million fragments mapped) values for the mRNAs and circRNAs were obtained for each sample, and RPM (reads per million) values for the miRNAs were calculated for each sample. The differentially expressed RNAs were analyzed using DESeq2 (v1.32.0) (http://www.bioconductor.org/packages/release/bioc/html/DESeq.html) [35], based on the *P* value calculated through multiple hypothesis testing [35]. Therefore, the following criteria were applied for the detection of differentially expressed RNAs: fold change > 2 and adjusted P value < 0.05 [36].

### Enrichment analysis

The differentially expressed circRNAs (DECs) (using the parent genes of DECs) were analyzed based on Gene Ontology (GO, http://www.geneontology.org/) terms and the Kyoto Encyclopedia of Genes and Genomes (KEGG, http://www.genome.jp/kegg/) using the clusterProfiler (10.1089/omi.2011.0118) package in R [37]. In this analysis, a cutoff value of 0.05 was used to filter the significantly enriched categories [38, 39]. Tests included multiple hypothesis testing based on the Benjamini-Hochberg method [35].

### Regulatory network analysis

The differentially expressed circRNAs, miRNAs and mRNAs were analyzed using miRanda [40]. A network of the interactions between ncRNAs and mRNAs was constructed using Cytoscape software [41]. The size of each node represents the degree, which is a parameter reflecting the number of nodes connected to a specific node. Therefore, significant nodes at the core areas of the regulated networks were considered highly associated with hypoxic adaptation.

### Altitude-dependent expression pattern analysis

Altitude-dependent variations were evaluated using STEM analysis [42] and different colors were used to indicate the significantly (*P*-values ≤0.05) enriched module profiles. Furthermore, a functional enrichment analysis was performed on all genes that showed similar tendencies in a module, as determined using g:Profiler [43] and the same parameter settings.

### Quantitative real-time (RT)-qPCR analysis

**Fig. 1:**
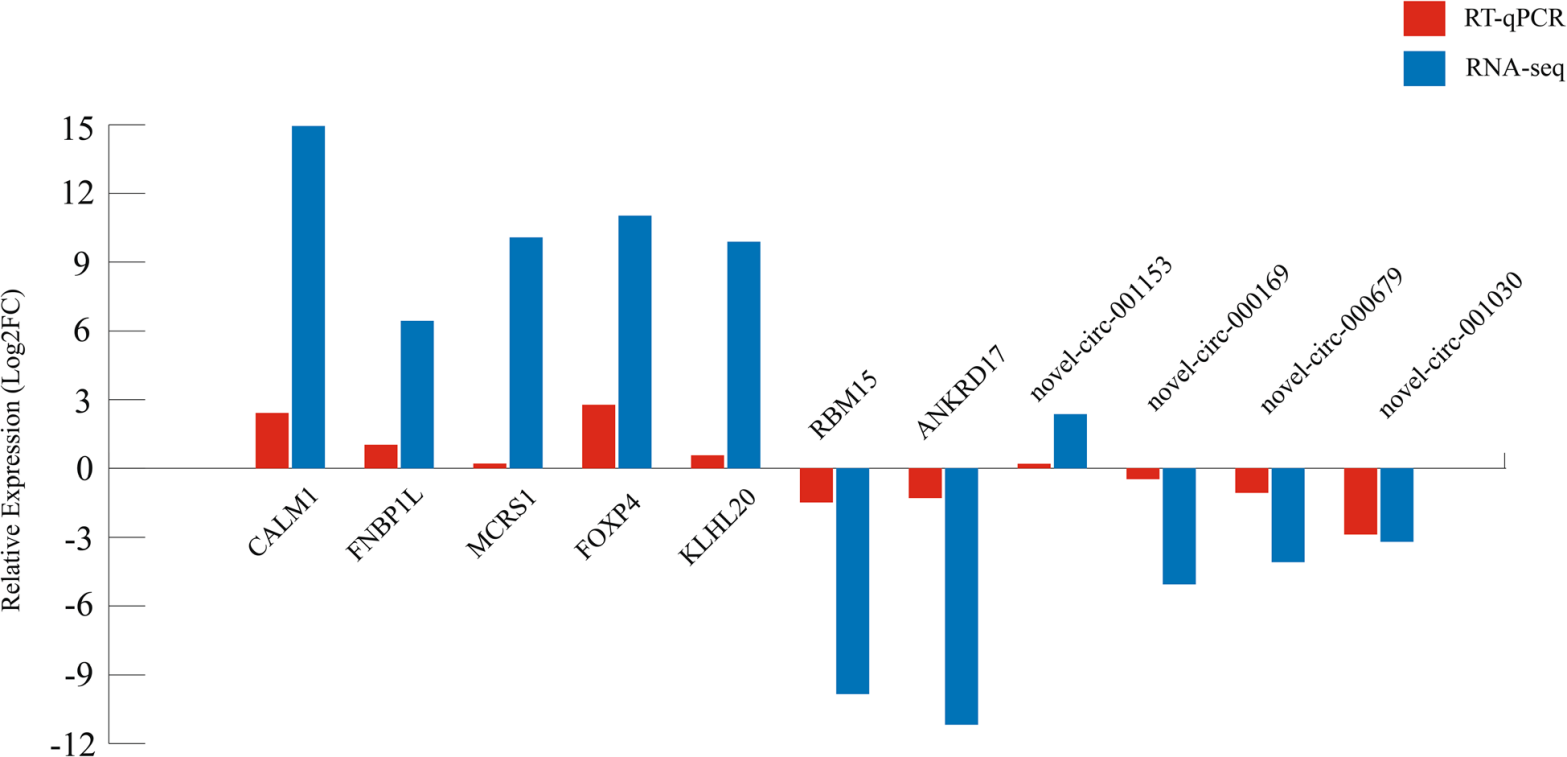
The RT-qPCR and RNA-seq methods were used to determine the expression levels of mRNA and circRNA. β-actin was designed as endogenous controls. The red and blue histograms represent the relative expression values determined by RT-qPCR and RNA-seq, respectively

Four DECs and seven DEGs were selected to verify the reliability of our data. Total RNA was extracted from lung tissue samples of yaks and cattle according to the manufacturer’s protocol (Invitrogen, Carlsbad CA, USA). qRT-PCR was used to test expression levels (Fig. 1). qRT-PCR was performed with a total volume of 20 μL containing 1 μL of each primer (10 μM), 1 μL of diluted cDNA and 10 μL of 2 × SYBR Premix ExTaq II (Takara, China). The cycling parameters for qRT-PCR amplification were 95 °C for 30 s followed by 40 cycles of 95 °C for 5 s and 60 °C for 30 s. Each qPCR experiment was performed in triplicate, and the 2^-∆∆Ct^ method was used to calculate the relative expression levels [44]. The primers for β-actin were designed as endogenous controls, and the amplified primers were listed in Supplementary Table S6. Moreover, the target specificity of the PCR primers was verified using BLAST, and the appearance of a single peak in the melting curve indicated the specificity of a primer. In summary, the qPCR expression trends indicated that the sequencing data were reliable (Fig. 1).

## Results

### Overview of RNA sequencing

To assess the mechanisms associated with genes involved in hypoxic adaptation, lung tissues were collected from yaks living at three different altitudes, and whole-transcriptome profiling of all mRNAs, noncoding RNAs (circRNAs) and microRNAs (miRNAs) was performed via high-throughput sequencing using Zaosheng cattle as a control. To obtain RNA sequencing (RNA-Seq) libraries, an average of 83.501 million clean reads were obtained from the 11 samples tested, and 62.99–91.49% of these reads were uniquely aligned to the reference genome using HiSAT2 [45]. At least 91.70% of the reads from all 11 samples presented values equal to or exceeding Q30 (Table S2). In addition, an average of 9.15 million clean reads were obtained from the small RNA-Seq libraries (Table S3). A total of 1000 known miRNAs and 3447 novel miRNAs were obtained after a series of analyses (Table S4).

**Fig. 2:**
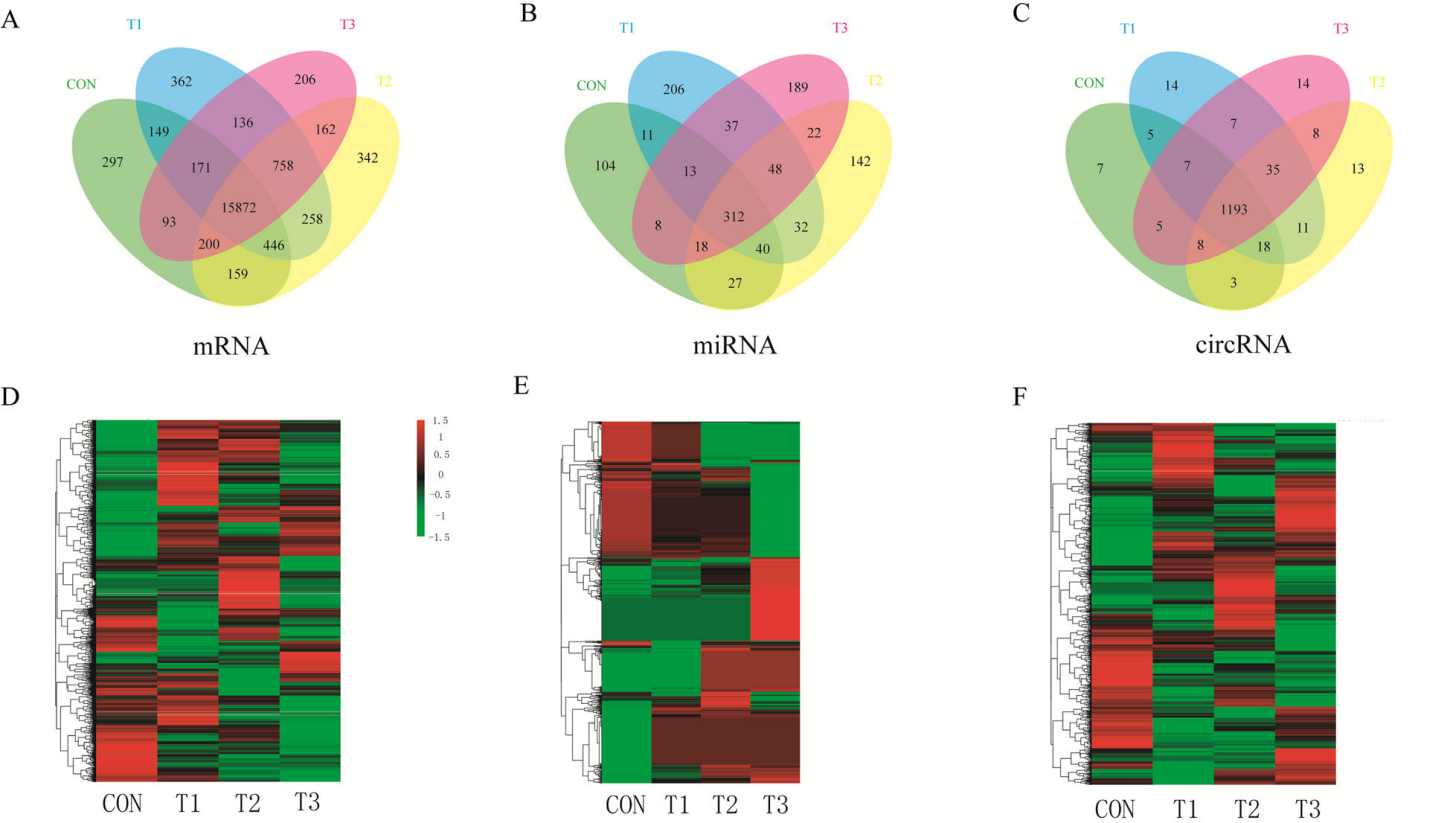
Comparative analysis of mRNAs, miRNAs, and circRNAs between yaks and cattle. Specific mRNAs (A), miRNAs (B), and circRNAs (C) shared between yaks and cattle. (D) Cluster analysis of differentially expressed mRNAs. (E) Cluster analysis of differentially expressed miRNAs. (F) Cluster analysis of differentially expressed circRNAs. The right color scale depicted the relative expression levels of mRNA, miRNA, or circRNA. The red colors indicate high relative expression; the green colors indicate low relative expression. The left panel shows the gene tree constructed based on a Pearson correlation analysis, and the value represents the gene expression values normalized with DES_EQ_2

In total, 21,764 mRNAs (Table S5) were obtained, and 15,872 of these mRNAs were generally expressed in the CON, T1, T2 and T3 groups. A total of 297 were specific to cattle, and 2224 were yak-specific mRNAs (Fig. 2A). Additionally, 1377 circRNAs were obtained; in addition, 7 and 102 of these circRNAs were specifically expressed in cattle and yak, respectively, and 1193 of these circRNAs were consistently expressed in the CON, T1, T2 and T3 groups (Fig. 2C). The analysis identified 4447 miRNAs, which included 1000 known and 3447 novel miRNAs (Table S5).

To identify the function of circRNAs in hypoxic adaptation, the profiles of circRNAs were investigated by high-throughput sequencing according to a previously described pipeline [32]. The expression abundance of circRNAs was measured based on TPM values, and the results indicated no abnormal expression in any of the 11 samples (Fig. S2). The results showed high consistency among the 11 tested samples, as shown in Fig. S2.

### Identification of altitude-dependent DEGs

The number and distribution of DEGs were identified from each pairwise comparison (Table 1). The number of DEGs was obtained from the comparison between yak and cattle were higher than from the comparison of yaks at different altitudes. In total, 275, 280 and 201 DEGs were identified from the comparison of yaks at different altitudes (Table 1). The DEGs obtained from the pairwise comparisons included more downregulated than upregulated genes. According to the same criteria, we also identified DECs: 26, 30 and eight DECs were obtained from comparison of yaks at different altitude gradients (Table 1).

**Table 1:** The number and distribution of differentially expressed mRNAs, circRNAs and miRNAs were identified from each pairwise comparison

Contrasts	mRNA		miRNA		circRNA	
	Up	Down	Up	Down	Up	Down
CON VS T1	1011	1019	19	12	69	52
CON VS T2	779	703	5	5	35	25
CON VS T3	691	772	16	18	44	27
T1 VS T2	117	158	11	11	18	8
T1 VS T3	120	160	6	11	14	16
T2 VS T3	119	82	23	21	6	2

CON group represent the lung tissue of Zaosheng cattle at an altitude of 1500 m

T1 group represent the lung tissue of yaks at an altitude of 3400 m

T2 group represent the lung tissue of yaks at an altitude of 4200 m

T3 group represent the lung tissue of yaks at an altitude of 5000 m

### Altitude-related gene expression profiles

**Fig. 3:**
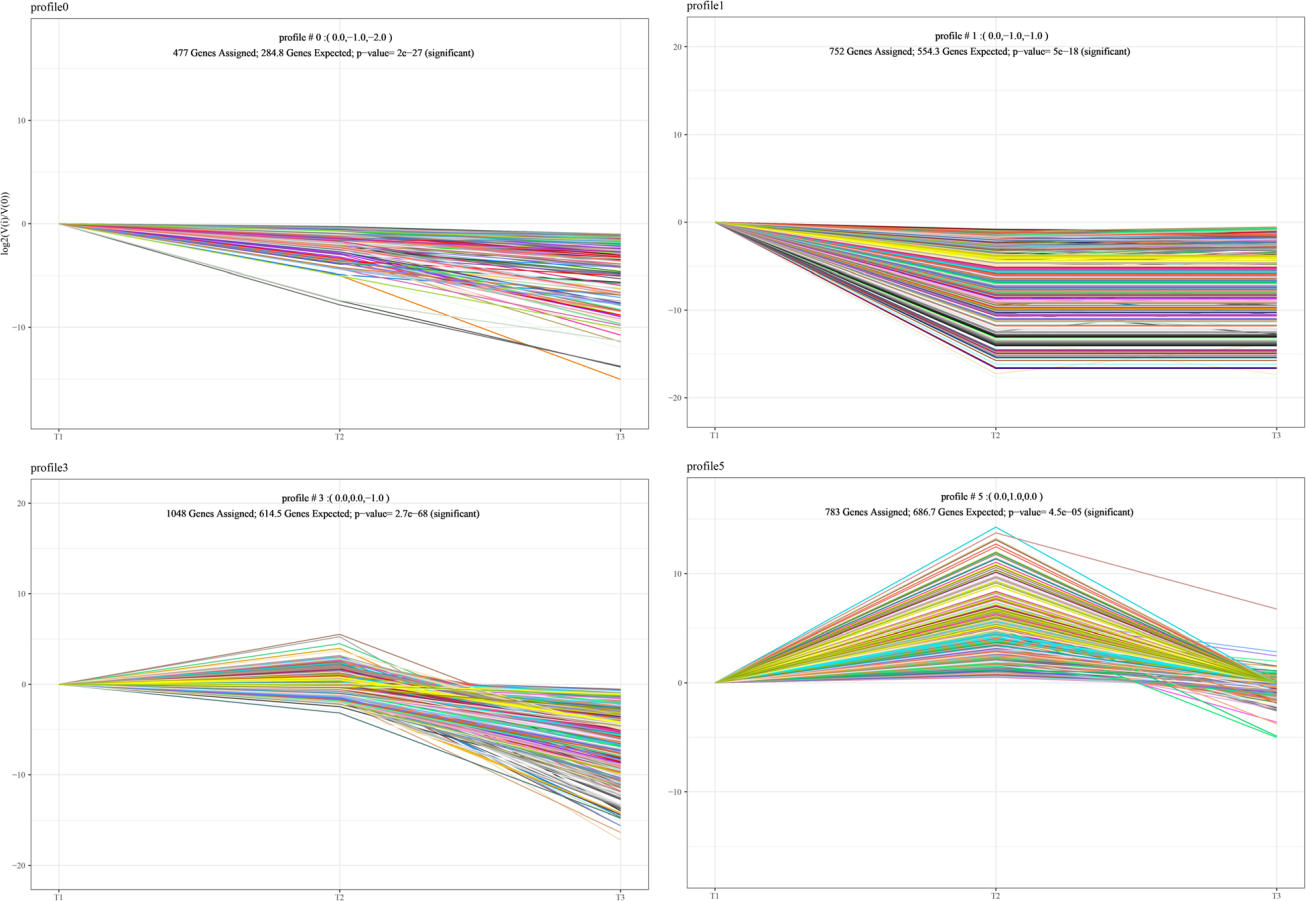
Profiles of altitude-dependent differentially expressed genes in yaks at different altitude gradients. The four modules show different expression patterns that were found to be significantly enriched via STEM analysis (nonsignificant modules are not shown)

DEGs of yaks at different altitude gradients were grouped into similar trends according to the STEM analysis. We adjusted the fold change threshold from 2 to 1.5 for more DEGs to enrich. We identified 13,424 DEGs and classified 4361 mRNAs into altitude-series expression patterns. The analysis of mRNAs revealed that four altitude profiles (*P* ≤ 0.05) were enriched by 3060 (70.17%) genes (Fig. 3). A functional enrichment analysis of the aforementioned genes that showed similar expression patterns in the four models was performed to investigate the potential functions of these clustered mRNAs. According to the altitude-related trend in gene expression, the expression patterns were divided into monotonically decreasing patterns and two other patterns with an inflection point (Fig. 3). The terms ‘microtubule-based process’ (GO:0007017) and ‘microtubule cytoskeleton organization’ (GO:0000226) were enriched in profile 3, and ‘channel activity’ (GO:0015267), ‘passive transmembrane transporter activity’ (GO:0022803) and ‘substrate-specific channel’ were enriched in profile 5.

### GO enrichment analysis of differentially expressed circRNAs

**Fig. 4:**
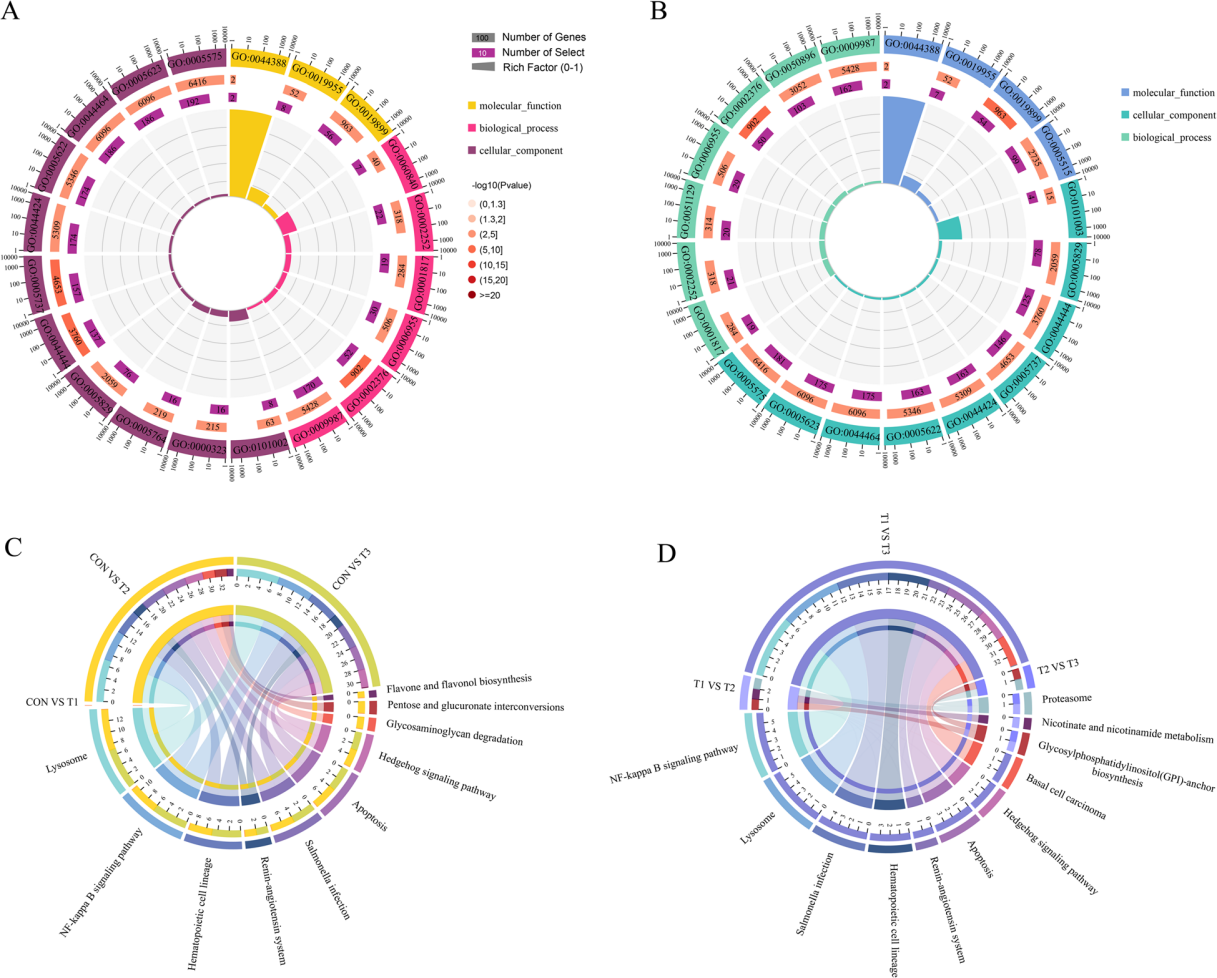
The KEGG and GO enrichment analyses of the differentially expressed circRNAs. GO enrichment analyses of the comparisons between yaks and cattle (**A**) and the comparisons among yaks at different altitude gradients (**B**). Enrichment cycle diagram including four circles, representing enrichment classification, the number of background genes in each categories and their Q or *P* values, the number of differentially expressed genes in the categories and the Rich Factor value of each category (The ratio of the number of differentially expressed genes to the number of background genes), respectively. KEGG enrichment analyses of the comparisons between yaks and cattle (**C**) and the comparisons among yaks at different altitude gradients (**D**). Each line represents a gene. OmicShare tools were used to visualize the GO and KEGG analysis results (http://www.omicshare.com/tools)

We performed GO enrichment analysis to investigate the biological roles of the DECs under high-altitude conditions. As demonstrated from the GO functional enrichment analysis, the DECs identified from the comparisons of yaks and cattle and those identified from the comparisons of yaks at different altitudes were commonly enriched in ‘immune system process’ within the biological process category, ‘cell’ in the cellular component category and ‘enzyme binding’ within the molecular function category (Fig. 4). The biological process ‘artery development’ and the cellular components ‘lytic vacuoles’ and ‘lysosomes’ were specific to the DECs identified from the comparisons of yaks and cattle (Fig. 4A). The biological processes ‘response to stimulus’ and ‘negative regulation of cellular component organization’ and the molecular function ‘protein binding’ were specifically enriched in the DECs obtained from the comparisons of yaks at different altitudes (Fig. 4B).

### Pathway enrichment analysis of differentially expressed circRNAs

In the KEGG pathway enrichment analyses, the DECs obtained from the comparisons of yaks and cattle and the comparisons of yaks at different altitudes were commonly enriched in the ‘NF-kappa B signaling pathway’ (Fig. 4). By comparing yaks and cattle, DECs were associated with metabolic pathways, such as ‘glycosaminoglycan degradation’ (Fig. 4C). By comparing yaks at different altitudes, which represent the effect of altitude changes on DECs, immune and cell cycle-related pathways were enriched, including ‘basal cell carcinoma’ (ko05217), ‘glycosylphosphatidylinositol (GPI)-anchor biosynthesis’ (ko00563), ‘nicotinate and nicotinamide metabolism’ (ko00760) and ‘proteasome’ (ko03050) (Fig. 4D).

### The differential RNA expression profiles between yak and cattle lungs identifies core regulatory networks and RNAs

We determined the mRNA and ncRNA (miRNA and circRNA) expression levels in yak and cattle lungs by high-throughput sequencing. Here, we aimed to uncover the biological processes and regulatory networks in yak lungs involved in hypoxic adaptation.

**Fig. 5:**
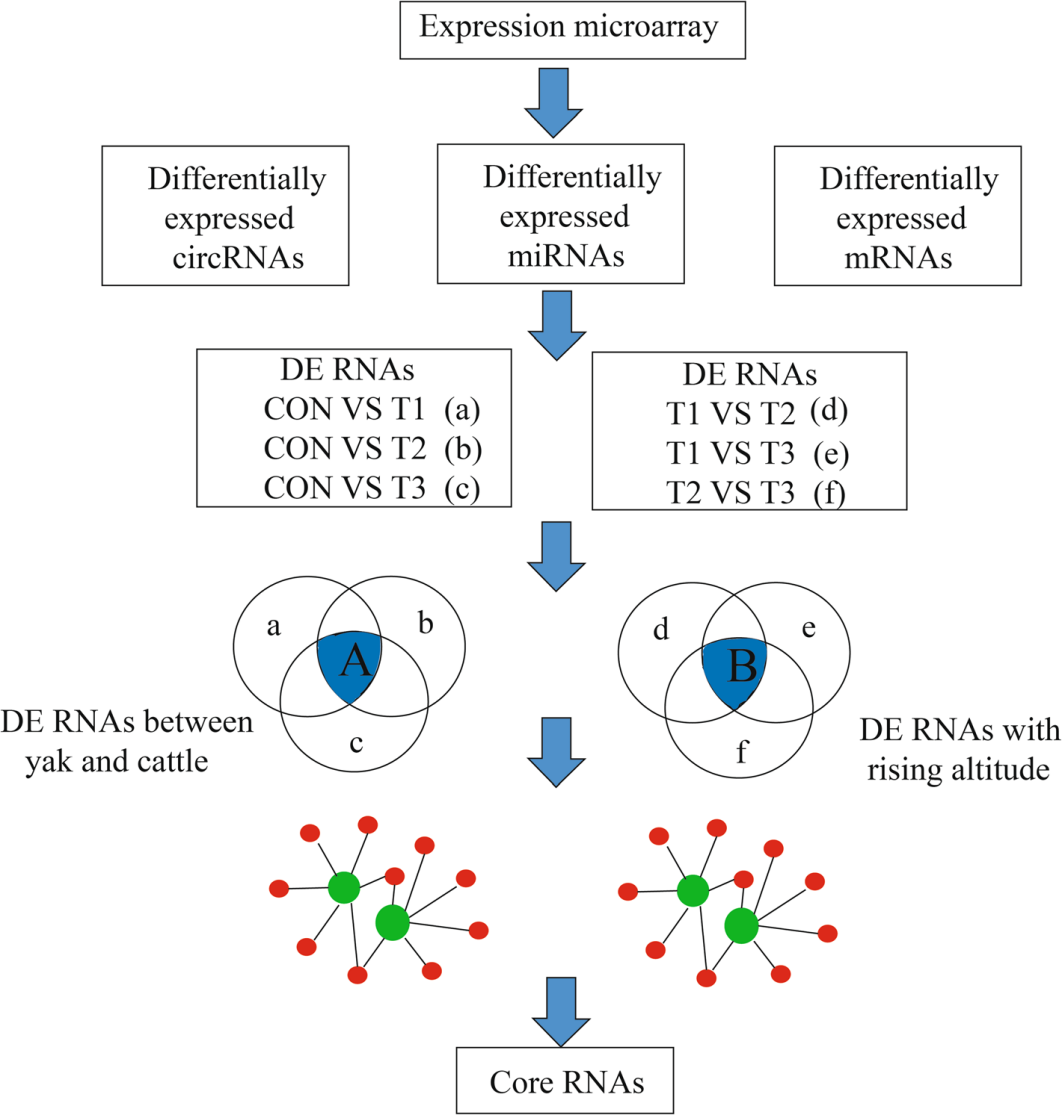
Experimental design for detecting key genes and regulatory molecules. Venn diagram indicating the intersection of the comparisons between yaks and cattle (part A) and the intersection of the comparisons among yaks at different altitude gradients (part B)

Key RNAs related to hypoxic adaptation were identified in Venn diagrams of differentially expressed RNAs (Fig. 5). The intersection of comparisons in CON vs. T1, CON vs. T2 and CON vs. T3 represented the main differences between yaks and cattle, and were considered the most interesting candidates (Fig. 5 part A).

**Fig. 6:**
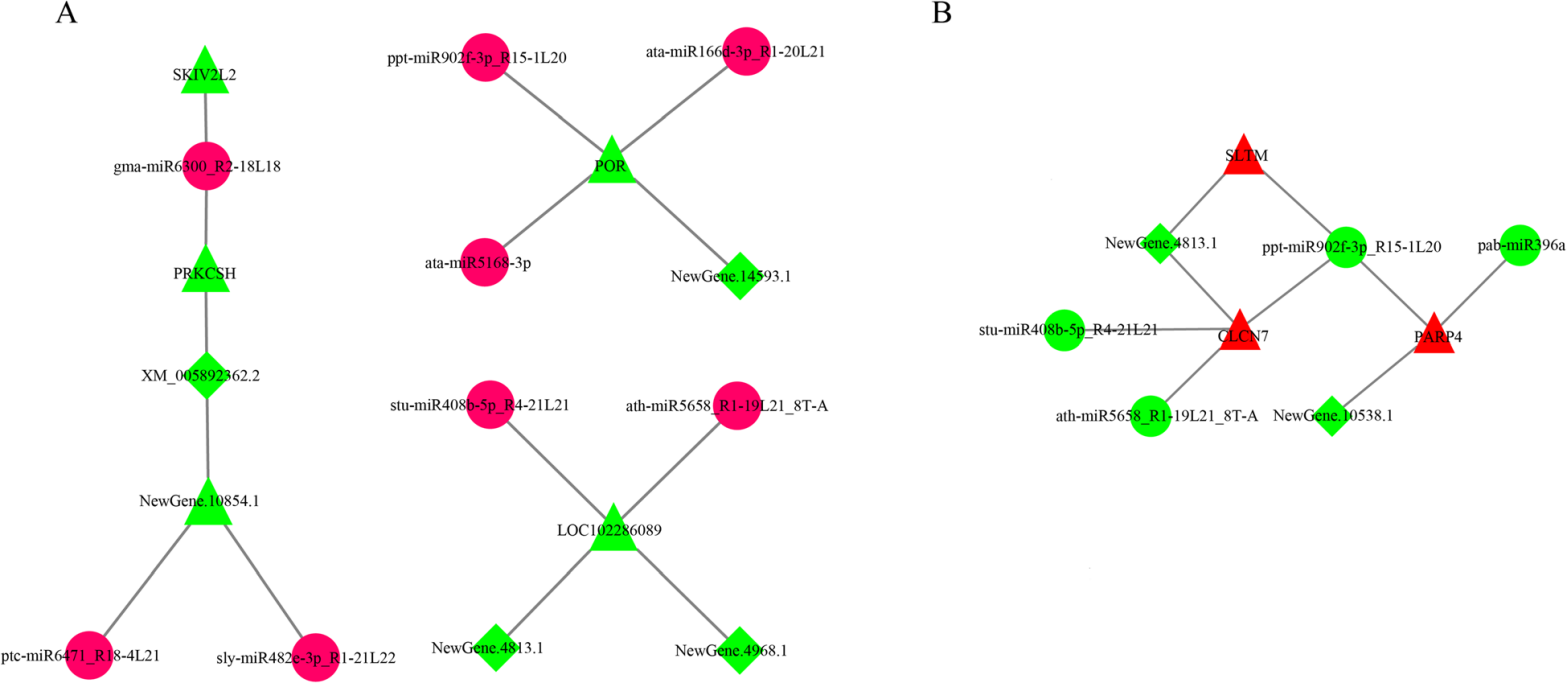
ceRNA coregulation network. The codifferentially expressed mRNAs, miRNAs and circRNAs in the intersection of the comparisons between yaks and cattle in the Venn diagram (part A) (A) and the intersection of the comparisons among yaks at different altitude gradients in the Venn diagram (part B) (B) were used to construct the ceRNA coregulation network. The triangular nodes represent mRNAs, the circular nodes represent miRNAs, and the rhombus-shaped nodes represent circRNAs. The red nodes represent upregulated transcripts, and the green nodes represent downregulated transcripts. The miRanda software and Cytoscape V3.2 software (http://cytoscape.org/) were used to analyzed and visualized network

Networks of differentially expressed RNAs were constructed, including mRNAs, miRNAs and circRNAs, at the intersection of the Venn diagrams. All miRNAs were upregulated, circRNAs and mRNAs were downregulated in the networks, including ppt-miR902f-3p_R15-1 L20, ata-miR166d-3p_R1-20L21, gma-miR6300_R2-18L18, ata-miR5168-3p, stu-miR408b-5p_R4-21L21, ptc-miR6471_R18-4 L21, sly-miR482e-3p_R1-21L22 and ath-miR5658_R1-19L21_8T-A, were identified as the most important RNAs between yaks and cattle, related circRNAs (XM_005892362.2, NewGene.14593.1, NewGene.4813.1 and NewGene.4968.1) and mRNAs (NewGene.1351.4, NewGene.7693.3, NewGene.10854.1, NewGene.11680.5 and NewGene.11562.2) identified by miRanda (Fig. 6).

### The differential RNA expression profiles among yak lungs at three different altitudes identifies core regulatory networks and RNAs

The intersection of comparisons in T1 vs. T2, T1 vs. T3 and T2 vs. T3 represented the main differences of yaks at different altitude gradient (Fig. 5 part B). Networks of differentially expressed RNAs were constructed at the intersection of the Venn diagrams, including ath-miR5658_R1–19L21_8T-A, pab-miR396a, stu-miR408b-5p_R4–21L21 and ppt-miR902f-3p_R15–1 L20, and their related circRNAs (NewGene.4813.1 and NewGene.10538.1) and mRNAs (NewGene.6186.6, XM_005886865.2 and NewGene.3754.1) (Fig. 6).

## Discussion

Yak, a domestic highland cattle breed native to China, has adapted to the hypoxic environment of the Tibetan Plateau [2]. In this study, we selected the lung, which is an important respiratory organ in animals, to analyze the hypoxic adaptation of yak. To identify the genes related to hypoxic adaptation, we compared yaks with cattle. Furthermore, compared yaks at different altitude gradients to explore the changes in genes related to hypoxia adaptation with increasing altitude.

We analyzed the obtained expression profiles of circRNAs to identify DECs related to hypoxic adaptation. We performed enrichment analyses of their target genes to explore the functions of DECs. The GO analysis revealed that the GO terms related to the DECs obtained from the comparison between yaks and cattle were enriched in the biological process ‘artery development’ (GO:0060840) and the cellular components ‘lytic vacuole’ (GO:0000323) and ‘lysosome’ (GO:0005764). The biological processes ‘response to stimulus’ (GO:0050896) and ‘negative regulation of cellular component organization’ (GO:0051129) and the molecular function ‘protein binding’ (GO:0005515) were specific to the DECs obtained from the comparison of yaks at different altitude gradients.

In contrast to the results obtained from the comparison of yaks living at different altitude gradients, the pathways related to the DECs obtained from the comparison between yaks and cattle were associated with metabolism and included ‘glycosaminoglycan degradation’, ‘pentose and glucuronate interconversions’ and ‘flavone and flavonol biosynthesis’. Glycosaminoglycan degradation and pentose and glucuronate interconversions are involved in glycan and carbohydrate metabolism, and pentose and glucuronate interconversions enhance sugar absorption and utilization. As one of the carbohydrate metabolism subcategories, the observed improvement in the pentose and glucuronate interconversion pathway is coincident with the fact that yaks need to metabolize more carbohydrates to survive in more complex environments. Previous studies have shown that yaks exhibit high energy metabolism, which allows their survival at high altitudes [2, 4]. Flavonoids can be used as aglycones or combined with sugars and/or organic acids [46, 47]. Flavones and flavonols belong to the group of phenolic compounds, and the function of polyphenols has been exploited, particularly with respect to cardiovascular diseases, metabolic syndrome, cancer, and obesity. Quercetin is the main representative flavonol, and the available evidence suggests that quercetin negatively regulates key signaling pathways associated with life-threatening diseases, including PI3K-AKT, MAPK, mTOR and NF-κB, and exhibits anticancer, anti-inflammatory, and cardiovascular protective properties [48, 49].

The analysis of the pathways specific to the DECs obtained from the comparison of yaks at three different altitudes revealed that metabolic pathways and immune and genetic information processing pathways were enriched. The enriched metabolic pathways included glycosylphosphatidylinositol (GPI)-anchor biosynthesis and nicotinamide and nicotinamide metabolism. GPIs act as membrane anchors for many cell surface proteins. The GPI anchoring of proteins is essential for mammalian embryogenesis, development, neurogenesis, fertilization, and the immune system [50–56]. Niacin, which is known as vitamin B3, is stable and can be transformed into nicotinamide in the body. Nicotinamide is involved in a variety of oxidative metabolic processes in the body, such as the oxidation process in tissue respiration and the anaerobic decomposition process of sugar. This metabolite is also involved in the regulation of energy metabolism, glucose and lipid metabolism, oxidative stress and inflammation [57, 58]. The immune and genetic information processing pathways are mainly related to cell carcinoma and the proteasome, respectively. Ultraviolet radiation (UV) is strong in plateau environments, and animal exposure to UV from sunlight causes basal cell carcinoma. The enrichment of this pathway indicates that UV exerts a significant effect on yaks. The proteasome, which is another enriched pathway, is the main complex related to specific protein degradation in eukaryotic cells, plays a key role in protein quality control and the maintenance of cell homeostasis and is the key factor in the adaptation of yaks to the Tibetan Plateau.

According to the experimental design, the key regulatory networks involved in hypoxia were identified based on the overlapping RNAs in the intersections in Venn diagrams (A and B in Fig. 5). The differentially expressed RNAs in part A were primarily related to hypoxic adaptation in yak, whereas those in part B were associated with changes in the regulatory mechanism of genes related to hypoxic adaptation at increasing altitude.

The core mRNAs in Venn diagrams part A (represented the differences between yaks and cattle) were predominantly involved in protein processing in the endoplasmic reticulum and fat deposition. The core mRNA PRKCSH (80 K-H) encodes hepatocystin [59]. Previous studies have shown that hepatocystin expression dominates the endoplasmic reticulum (ER) [60] and acts as the noncatalytic β-subunit of glucosidase II. Therefore, lack of hepatocystin reduces the activity of glucosidase II, which is essential for the processing and folding of glycoproteins in the ER [61]. Defects in protein glycosylation and folding trigger the unfolded protein response (UPR), which is a cellular stress response related to the ER. The accumulation of misfolded proteins activates the UPR, which restores normal cellular function through the degradation of misfolded proteins and activation of the production of chaperones, such as glucose-regulated protein 78 (GRP78) [62, 63]. Hypoxia might induce cell apoptosis under severe ER stress, and downregulated PRKCSH amplifies the pro-survival UPR signal, which Reduces damage associated with ER stress. In addition, some studies have indicated that the Inhibition of hepatocystin alone might not be enough to activate the UPR, while inhibition of hepatocystin under hypoxic conditions can activate the UPR [64]. The mRNA enrichment analysis revealed that the pathway ‘protein processing in the endoplasmic reticulum (ER)’ was also enriched, which suggests that this pathway plays an indelible role in the adaptation of yaks to resist hypoxia in the plateau.

**Fig. 7:**
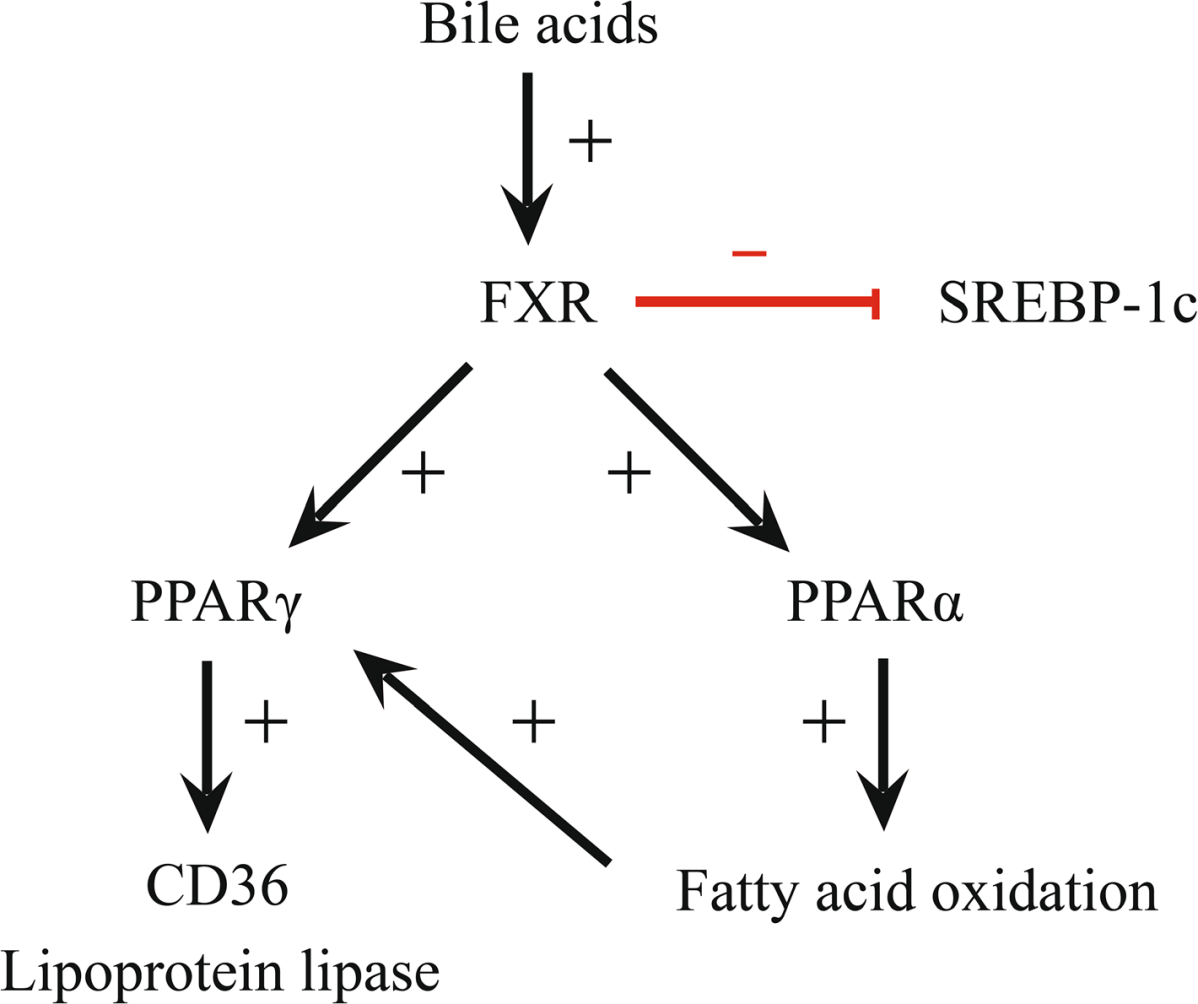
POR influences the regulatory mechanism of fat metabolism by regulating PPAR

NADPH-cytochrome P450 oxidoreductase (POR) plays an important roles in the biotransformation of xenobiotics and the biosynthesis and metabolism of many endogenous substances such as cholesterol, steroid hormones, bile acids and vitamins. POR has the ability to donate electrons to multiple acceptors and is necessary for the synthesis of cholesterol [65] and bile acids. Furthermore, POR play an important role in heme catabolism, synthesis of steroid hormone and the activation of vitamin D [66]. In Por-null cells, cholesterol cannot be catabolized to bile acids, which suggests that oxysterols might be formed and accumulate in these cells. In addition, oxysterols activate the liver X receptor (LXR), stimulates transcription of sterol regulatory element-binding protein-1c (SREBP-1c), thereby induces fatty acid and triglyceride synthesis [67]. Farnesoid X receptor (FXR) activation suppresses SREBP-1c transcription; thus, this suppression is decreased in the absence of bile acids (the natural activators of FXR) (Fig. 7). In Por-null cells, increased expression of the following two genes is related to lipid uptake: lipoprotein lipase and CD36 [68]. The peroxisome proliferator-activated receptor PPARα down-regulates the fatty acid catabolism. PPARα expression is significantly reduced in Por-null cells resulting in a reduction in the oxidation of fatty acids [68]. Since PPARα expression is regulated by FXR [69], the lack of FXR signaling might lead to a decrease in PPARα expression, thereby reduce fatty acid catabolism. The resulting increase in the fatty acid content might induce PPARγ expression and thereby lead to increased expression of genes related to fatty acid uptake (lipoprotein lipase and CD36) and increased fat deposition (Fig. 7) [68]. The mRNA enrichment analysis between yaks and cattle revealed that GO terms related to ‘protein targeting to peroxisome’ and ‘long-chain fatty acid metabolic process’ were also enriched. This finding led us to speculate that the mechanism of fat deposition through POR regulates PPAR, which allows yaks to resist the cold condition on the plateau.

The core mRNAs in Venn diagrams part B (represented the differences among yaks at different altitude gradients) were significant enriched in the maintenance of normal cellular biological functions.

Solar radiation is an important and inevitable component of the plateau environment. Ultraviolet (UV) radiation damages many cellular components, such as DNA. UV-damaged DNA is repaired by light repair and dark repair (NER) [70, 71]. NER consists of two sub-pathways: global genomic repair (GGR) recognizes UV damage in DNA throughout the genome, and transcription-coupled repair (TCR) initiates the repair of transcribed strands. In mammals, UVSSA has been implicated in TCR. Loss-of-function alleles in UVSSA exhibit higher UV sensitivity [72]. The core gene UVSSA might play an important role in the lung tissue of yaks to help the animals overcome UV radiation. The results from the previous enrichment analysis of circRNAs revealed the huge impact of UV radiation on yaks. The resistance of yaks to UV radiation is an important part of their adaptation on the Tibetan Plateau.

Other core genes in the network obtained from the comparison of yaks at different altitudes, such as SAFB-like transcriptional modulator (SLTM) and PARP4, were also related to sustained life activities of cells. SLTM has well-described functions in transcriptional repression and RNA splicing, is ubiquitously expressed and plays important roles in numerous cellular processes, including cell growth, DNA repair, stress response, and apoptosis. PARP4 plays an active role in cellular recovery from DNA damage, improves genetic stability and reduces recombination. In contrast, PARP4 also makes cells insensitive to apoptotic stimuli [73]. In addition, the core mRNAs in part B include CLCN7. The CLCN7 gene encodes the 803-amino-acid protein chloride channel protein 7 (ClC-7), which provides the chloride conductance needed for efficient proton pumping in the osteoclast ruffled membrane [74] and is involved in acidification of the resorption lacuna. Mutations in the CLCN7 gene result in autosomal dominant osteoporosis.

## Conclusion

Our genomic data have provided important insights into the adaptation of yaks to high altitude. This phenomenon can be further elucidated through a functional analysis of genes identified as signs of adaptive evolution from comparative studies between yaks and other animals exposed to high altitude-related stress. The identification of genes needed for natural adaptation to high altitude might contribute to improvements in the treatment, understanding and prevention of altitude sickness and other hypoxia-related diseases in humans.

## Supplementary Information

**Additional file 1.** Fig. S1. Distribution of sampling location. T1 represented the yaks from Maqu County in the Gannan Tibetan Autonomous Prefecture of Gansu Province at an altitude of 3400 m, T2 represented the yaks from Bange County, Linzhou County and Dangxiong County in the Tibetan Autonomous Region at an altitude of 4200 m, T3 represented the yaks from Anduo County in the Tibetan Autonomous Region at an altitude of 5000 m, and CON represented the Zaosheng cattle from Ningxian County in Gansu Province at an altitude of 1500 m. Fig. S2. (A) Expression levels of circRNAs. The lines of the whiskers in the box represent the medians. (B) Density distribution of circRNAs. (C) Percentage of expression levels of circRNAs in each group. Table S1. Characteristics of samples. Table S2. Summary of quality and statistics of the mRNA data. Table S3. Summary of quality and statistics of the miRNA data. Table S4. Distribution of known and novel miRNAs in each sample. Table S5. Classification of mRNAs, miRNAs and circRNAs. Table S6. Primers designed for qRT-PCR validation of candidate circRNAs and mRNAs.

## Data Availability

All raw and processed sequencing data generated in this study have been submitted to the NCBI GEO (https://www.ncbi.nlm.nih.gov/geo/) database under accession number GEO: GSE153956 and GSE153962.

## Abbreviations

DECs: differentially expressed circRNAs
DEGs: Differentially expressed gene
GPI: Glycosylphosphatidylinositol
UV: Ultraviolet radiation
ER: Endoplasmic reticulum
UPR: Unfolded protein response
POR: NADPH-cytochrome P450 oxidoreductase
GRP78: Glucose-regulated protein 78
LXR: Liver X receptor
SREBP-1c: Sterol regulatory element-binding protein-1c
FXR: Farnesoid X receptor
PPARα: Peroxisome proliferator-activated receptor α
PPARγ: Peroxisome proliferator-activated receptor γ
NER: Nucleotide excision repair
GGR: Global genomic repair
TCR: Transcription-coupled repair
SLTM: SAFB-like transcriptional modulator
ClC-7: 803-amino-acid protein chloride channel protein 7
