# Supplementary material for: A comparative analysis of differentially expressed mRNAs, miRNAs and circRNAs provides insights into the key genes involved in the high-altitude adaptation of yaks
Source: BMC Genomics. 2021 Oct 15;22:744. doi: 10.1186/s12864-021-08044-9 (PMC8518315; doi:10.1186/s12864-021-08044-9)
Supplement: Supplementary file 1 — Additional file 1. Fig. S1. Distribution of sampling location. T1 represented the yaks from Maqu County in the Gannan Tibetan Autonomous Prefecture of Gansu Province at an altitude of 3400 m, T2 represented the yaks from Bange County, Linzhou County and Dangxiong County in the Tibetan Autonomous Region at an altitude of 4200 m, T3 represented the yaks from Anduo County in the Tibetan Autonomous Region at an altitude of 5000 m, and CON represented the Zaosheng cattle from Ningxian County in Gansu Province at an altitude of 1500 m. Fig. S2. (A) Expression levels of circRNAs. The lines of the whiskers in the box represent the medians. (B) Density distribution of circRNAs. (C) Percentage of expression levels of circRNAs in each group. Table S1. Characteristics of samples. Table S2. Summary of quality and statistics of the mRNA data. Table S3. Summary of quality and statistics of the miRNA data. Table S4. Distribution of known and novel miRNAs in each sample. Table S5. Classification of mRNAs, miRNAs and circRNAs. Table S6. Primers designed for qRT-PCR validation of candidate circRNAs and mRNAs. [file 12864_2021_8044_MOESM1_ESM.docx]

**Supplementary materials**


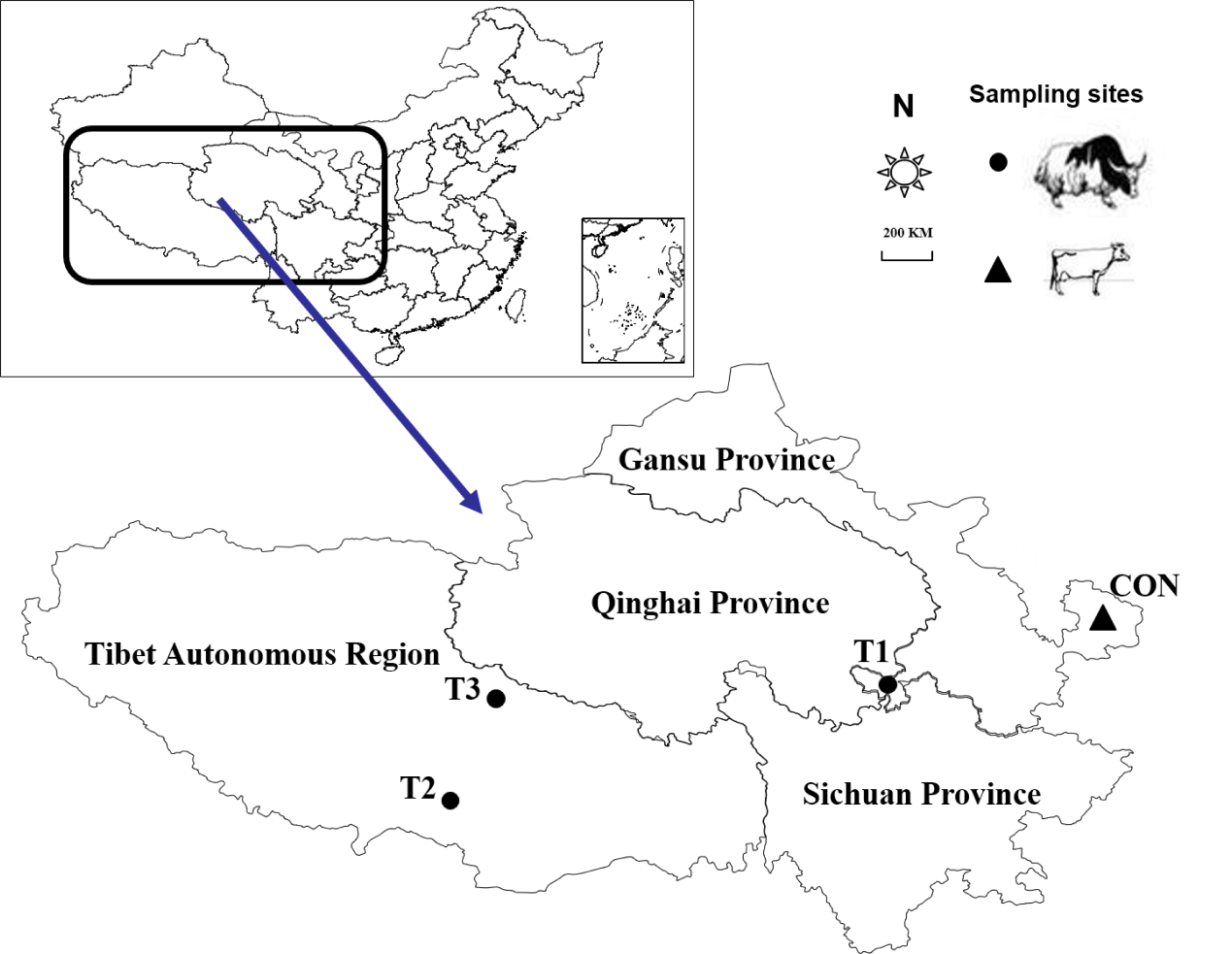


Figure S1. Distribution of sampling location. T1 represented the yaks from Maqu County in the Gannan Tibetan Autonomous Prefecture of Gansu Province at an altitude of 3,400 m, T2 represented the yaks from Bange County, Linzhou County and Dangxiong County in the Tibetan Autonomous Region at an altitude of 4,200 m, T3 represented the yaks from Anduo County in the Tibetan Autonomous Region at an altitude of 5,000 m, and CON represented the Zaosheng cattle from Ningxian County in Gansu Province at an altitude of 1,500 m.


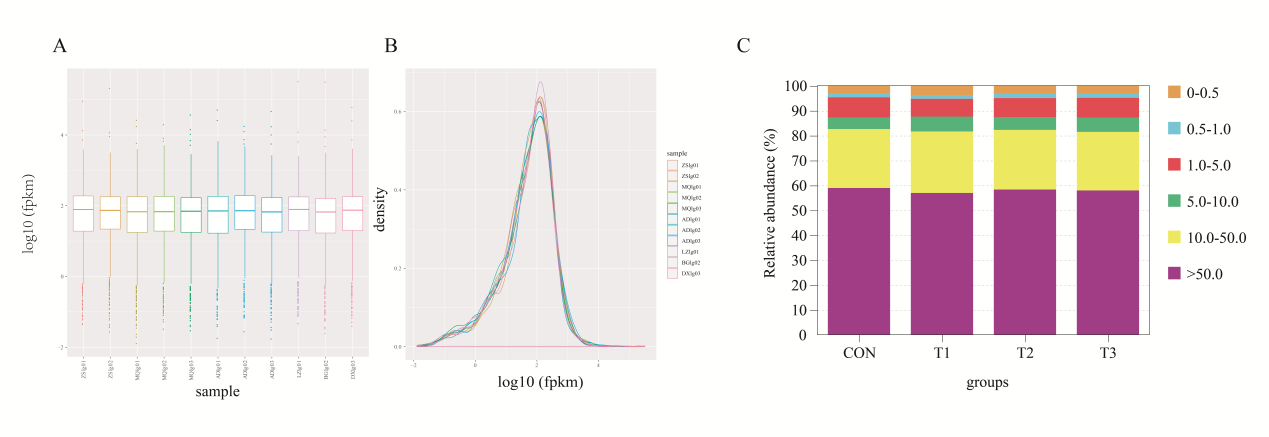


Figure S2. (A) Expression levels of circRNAs. The lines of the whiskers in the box represent the medians. (B) Density distribution of circRNAs. (C) Percentage of expression levels of circRNAs in each group.

Table S1. Characteristics of samples.

| Sample | Category | Sample size | Sampling site | Altitude (m) | Duplicates |
| --- | --- | --- | --- | --- | --- |
| CON | Zaosheng cattle | 2 | Ningxian, Gansu, China | 1,500 | ZSlg01 |
|  |  |  |  |  | ZSlg02 |
| T1 | Yak | 3 | Maqu, Gannan Tibetan Autonomous Prefecture, Gansu, China | 3,400 | MQlg01 |
|  |  |  |  |  | MQlg02 |
|  |  |  |  |  | MQlg03 |
| T2 | Yak | 3 | Dangxiong, Linzhou and Bange, Tibet Autonomous Region, China | 4,200 | LZlg01, |
|  |  |  |  |  | BGlg02 |
|  |  |  |  |  | DXlg03 |
| T3 | Yak | 3 | Anduo, Tibetan Autonomous Region, China | 5,000 | ADlg01 |
|  |  |  |  |  | ADlg02 |
|  |  |  |  |  | ADlg03 |

Table S2. Summary of quality and statistics of the mRNA data.

| Sample | Duplicates | Raw reads (M) | Clean reads (M) | Clean bases (G) | Q20 (%) | Q30 (%) | Total mapped | Uniquely mapped |
| --- | --- | --- | --- | --- | --- | --- | --- | --- |
| CON | ZSlg01 | 74.864 | 73.483 | 10.958(97.6%) | 10.759(98.2%) | 10.337(94.3%) | 65.162 | 65.162(88.68%) |
|  | ZSlg02 | 78.358 | 76.714 | 11.422(97.2%) | 11.079(97.0%) | 10.485(91.8%) | 66.666 | 66.666(86.90%) |
| T1 | MQlg01 | 93.487 | 92.431 | 13.782(98.3%) | 13.570(98.5%) | 13.091(95.0%) | 80.296 | 80.296(86.87%) |
|  | MQlg02 | 91.873 | 91 | 13.587(98.6%) | 13.408(98.7%) | 12.997(95.7%) | 82.703 | 82.703(90.88%) |
|  | MQlg03 | 69.43 | 68.33 | 10.197(97.9%) | 10.018(98.2%) | 9.639(94.5%) | 62.38 | 62.380(91.29%) |
| T2 | LZlg01 | 82.151 | 81.188 | 12.098(98.2%) | 11.907(98.4%) | 11.485(94.9%) | 72.621 | 72.621(89.45%) |
|  | BGlg02 | 71.79 | 70.376 | 10.476(97.3%) | 10.161(97.0%) | 9.611(91.7%) | 64.234 | 64.234(91.27%) |
|  | DXlg03 | 80.375 | 79.419 | 11.826(98.1%) | 11.633(98.4%) | 11.208(94.8%) | 71.896 | 71.896(90.53%) |
| T3 | ADlg01 | 88.463 | 87.25 | 12.995(97.9%) | 12.788(98.4%) | 12.335(94.9%) | 68.698 | 68.698(78.74%) |
|  | ADlg02 | 87.835 | 85.665 | 12.739(96.7%) | 12.531(98.4%) | 12.098(95.0%) | 53.963 | 53.963(62.99%) |
|  | ADlg03 | 113.71 | 112.659 | 16.789(98.4%) | 16.583(98.8%) | 16.107(95.9%) | 103.072 | 103.072(91.49%) |

ZSlg01 and ZSlg02 indicate libraries derived from the lung tissue of Zaosheng cattle in two biological replicates.

MQlg01, MQlg02, and MQlg03 indicate libraries derived from the lung tissue of yaks living at an altitude 3,400 m in three biological replicates.

LZlg01, BGlg02, and DXlg03 indicate libraries derived from the lung tissue of yaks living at an altitude 4,200 m in three biological replicates.

ADlg01, ADlg02, and ADlg03 indicate libraries derived from the lung tissue of yaks living at an altitude 5,000 m in three biological replicates.

These designations are used similarly hereafter.

Table S3. Summary of quality and statistics of the miRNA data.

| Sample | Duplicates | Raw Reads(M) | Raw Bases(G) | Raw Q20(G) | Raw Q30(G) | Clean Reads(M) | Clean Bases(G) | Clean Q20(G) | Clean Q30(G) | Average Length(bp) |
| --- | --- | --- | --- | --- | --- | --- | --- | --- | --- | --- |
| CON | ZSlg01 | 8.908 | 1.336 | 0.931(69.7%) | 0.810(60.6%) | 8.226(92.3%) | 0.180(13.4%) | 0.179(99.8%) | 0.178(99.1%) | 21.8 |
|  | ZSlg02 | 9.282 | 1.402 | 1.114(79.5%) | 0.963(68.7%) | 8.260(89.0%) | 0.182(13.0%) | 0.181(99.9%) | 0.181(99.7%) | 22 |
| T1 | MQlg01 | 9.283 | 1.402 | 1.123(80.1%) | 0.981(70.0%) | 9.066(97.7%) | 0.197(14.1%) | 0.197(99.9%) | 0.197(99.8%) | 21.8 |
|  | MQlg02 | 10.633 | 1.606 | 1.253(78.1%) | 1.085(67.6%) | 8.646(81.3%) | 0.189(11.8%) | 0.189(99.9%) | 0.188(99.7%) | 21.8 |
|  | MQlg03 | 9.917 | 1.497 | 1.190(79.5%) | 1.032(68.9%) | 8.899(89.7%) | 0.196(13.1%) | 0.196(99.9%) | 0.195(99.6%) | 22 |
| T2 | LZlg01 | 13.171 | 1.989 | 1.591(80.0%) | 1.380(69.4%) | 12.474(94.7%) | 0.271(13.6%) | 0.270(99.9%) | 0.269(99.6%) | 21.7 |
|  | BGlg02 | 6.713 | 1.007 | 0.704(69.9%) | 0.615(61.1%) | 6.226(92.7%) | 0.138(13.7%) | 0.138(99.8%) | 0.137(99.2%) | 22.2 |
|  | DXlg03 | 10.239 | 1.546 | 1.223(79.1%) | 1.059(68.5%) | 9.069(88.6%) | 0.199(12.9%) | 0.199(99.9%) | 0.199(99.7%) | 22 |
| T3 | ADlg01 | 11.611 | 1.753 | 1.407(80.2%) | 1.221(69.6%) | 11.315(97.4%) | 0.246(14.0%) | 0.246(99.9%) | 0.245(99.6%) | 21.7 |
|  | ADlg02 | 10.935 | 1.651 | 1.324(80.2%) | 1.157(70.0%) | 10.633(97.2%) | 0.233(14.1%) | 0.232(99.9%) | 0.232(99.7%) | 21.9 |
|  | ADlg03 | 9.257 | 1.398 | 1.098(78.5%) | 0.953(68.2%) | 7.850(84.8%) | 0.172(12.3%) | 0.172(99.9%) | 0.171(99.7%) | 21.9 |

Table S4. Distribution of known and novel miRNAs in each sample.

| miRNA | ZSlg01 | ZSlg02 | MQlg01 | MQlg02 | MQlg03 | LZlg01 | BGlg02 | DXlg03 | ADlg01 | ADlg02 | ADlg03 |
| --- | --- | --- | --- | --- | --- | --- | --- | --- | --- | --- | --- |
| known_miRNA | 29 | 107 | 92 | 112 | 111 | 88 | 20 | 117 | 110 | 107 | 107 |
| novel_miRNA | 334 | 303 | 292 | 335 | 331 | 409 | 341 | 260 | 258 | 281 | 303 |

Table S5. Classification of mRNAs, miRNAs and circRNAs.

| class | known | novel | total |
| --- | --- | --- | --- |
| circRNA | 540 | 837 | 1377 |
| mRNA | 11749 | 10015 | 21764 |
| miRNA | 1000 | 3447 | 4447 |
| Total | 13289 | 14299 | 27588 |

Table S6. Primers designed for qRT-PCR validation of candidate circRNAs and mRNAs.

| Genes | GenBank accession No. | Primer sequences (5'-3') | Product size/bp |
| --- | --- | --- | --- |
| CALM1 | XM_005888605.2 | F: GCACCATCACAACCAAGGAAC | 176 |
|  |  | R: CTCTTCACTGTCGGTGTCTTTCA |  |
| FNBP1L | XM_005894694.1 | F: AAGAGCCAAGGTTTACTTCGTGT | 86 |
|  |  | R: TGCTACTACTTCTCGCTGTCCTG |  |
| MCRS1 | XM_014478414.1 | F: TTCCGACGCAGAGGACTTG | 150 |
|  |  | R: TCTACCAGCACCTGCCACTTAT |  |
| FOXP4 | XM_005896443.2 | F: AGGATGTTCGCCTATTTCCG | 140 |
|  |  | R: TTCTGGTACTCCCGCTCGTC |  |
| KLHL20 | XM_014476724.1 | F: GGACAAGACCACGGAAACCTA | 127 |
|  |  | R: GCTACCATTCGCCATTCATTAG |  |
| RBM15 | XM_005891280.2 | F: AGTGGAGGACGGATTGTTTCA | 109 |
|  |  | R: GTTCACAAAGGCTACTCGCTCA |  |
| ANKRD17 | XM_005891242.2 | F: TGTTAGTTGTGCGTTGGATGAA | 109 |
|  |  | R: TGAACAGGCTTCTGCCAAACT |  |
| novel-circ-001153 | XM_005895691.1 | F: TGGTGGATGGTGTTCGC | 75 |
|  |  | R: GTGTAAGTCAAGAGGGAAGTCATT |  |
| novel-circ-000169 | XM_005888274.2 | F: AGGCATGACCCAGAACTGA | 258 |
|  |  | R: GGAATTTCATCTGTTTGGCTC |  |
| novel-circ-000679 | XM_005891846.2 | F: GGGAGATGAGATGGACCTGG | 253 |
|  |  | R: ACCAGCCGACTCAGGGAAGT |  |
| novel-circ-001030 | XM_005894440.2 | F: CCATACACCCTCTTCACCCA | 120 |
|  |  | R: AAACTTCTCGCTGCTGCTCT |  |
